# An App to Help Young People Self-Manage When Feeling Overwhelmed (ReZone): Protocol of a Cluster Randomized Controlled Trial

**DOI:** 10.2196/resprot.7019

**Published:** 2017-11-03

**Authors:** Julian Edbrooke-Childs, Jaime Smith, Jessica Rees, Chloe Edridge, Ana Calderon, Felicity Saunders, Miranda Wolpert, Jessica Deighton

**Affiliations:** ^1^ Evidence Based Practice Unit University College London and Anna Freud National Centre for Children and Families London United Kingdom; ^2^ Anna Freud National Centre for Children and Families London United Kingdom

**Keywords:** cluster trial, behavioral difficulties, schools

## Abstract

**Background:**

The association between behavioral difficulties and academic attainment is well established. Recent policy advising schools on managing behavior has promoted the early identification of behavioral difficulties. There is also increasing research into mHealth interventions to provide support for emotional and behavioral difficulties for young people.

**Objective:**

The primary aim of the proposed research is to examine the effectiveness of an mHealth intervention, ReZone, in reducing emotional and behavioral difficulties in young people.

**Methods:**

The protocol is a cluster trial of 12 classes with N=120 students with classes randomized to ReZone or management as usual. Multilevel modeling will be used to compare ReZone versus management as usual accounting for classroom-level variation.

**Results:**

Baseline data collection started in February 2017 and ended in April 2017. Follow-up data collection started in April 2017 and ended in June 2017.

**Conclusions:**

The proposed research will provide evidence as to whether ReZone is effective at helping young people to self-manage when feeling overwhelmed.

**Trial Registration:**

ISRCTN 13425994; http://www.isrctn.com/ISRCTN13425994 (Archived by WebCite at http://www.webcitation.org/6tePwwiHk)

## Introduction

### Background

In England, between 3% and 7% of school-aged children experience behavioral difficulties [1]. The association between behavioral difficulties and academic attainment is well established [2]. In particular, increasing levels of behavioral difficulties have been shown to predict negative change in academic attainment [3], and a systematic review showed a consistent association between behavioral difficulties and early school leaving [4]. Correspondingly, recent policy advising schools on managing behavior has promoted the early identification of behavioral difficulties [5]. Behavioral difficulties may interfere with school engagement, and local government bodies in England are responsible for providing alternative provision schools. Alternative provision is defined as “education arranged by local authorities for pupils who, because of exclusion, illness or other reasons, would not otherwise receive suitable education; education arranged by schools for pupils on a fixed period of exclusion; and pupils being directed by schools to off-site provision to improve their behavior” [6].

A range of programs exist to support social and emotional aspects of learning (SEAL) in students, with a systematic review identifying 113 school interventions and 222 out-of-school interventions in the United Kingdom [7]. SEAL interventions are centered on reducing risk factors and fostering protective mechanisms and focus on the development of 5 essential skills and competencies: self-awareness, self-management, social awareness, relationship skills, and responsible decision making [8]. A review identified that the majority of children targeted for these interventions were aged 5 to 10 years (56%) and were classroom-based (74%) [9]. A review of meta-analyses of SEAL interventions concluded that high-quality SEAL programs can successfully increase children’s socioemotional and language skills, along with fostering positive outcomes and preventing negative ones [10]. When compared to other youth development programs offered to school-age youth, SEAL programs are among the most successful [11].

### Technology-Enabled Mental Health Care

In terms of mental health, delivering services using new technologies is a developing area of interest in the English government, which recommends the increased use of technology-enabled interventions to improve access to services [12]. This trend is particularly relevant to young people, a group with disproportionate levels of mental health problems and the highest use of technology. For example, 8 in 10 children aged 12 to 15 years have access to a mobile phone, with the majority (59%) using them to access the Internet. The proportion of children aged 5 to 10 years having access to a tablet computer has increased from 51% to 71% in the past 3 years [13]. Correspondingly, there has been an increase in the use of technology (eHealth) and mobile and wireless technologies (mHealth) for health care promotion and service delivery.

Technology-enabled health care is “not just about technology, but about empowering patients to exercise greater choice and control” [12] through increasing their sense of agency [14]. Some argue that it results in patients having a better understanding of their conditions [15]. Mobile phone apps particularly enable real-time recording of moods, behaviors, and activities, which can then be shared with others to improve engagement [16]. The use of mobile phone and tablet technology for service provision has a range of potential benefits, including the ability to record information, portability, acceptability, relatively low costs, and near constant connectivity to young people and carers [17]. In addition, apps provide real-life instruction to enable self-management (eg, breathing techniques like Breathe 2 Relax [18]).

There is increasing research into mHealth interventions [19,20]. One study highlighted limitations in the security of apps in a health library [21]. A systematic review of youth mental health interventions via mobile phones [22] discovered key gaps in the literature in terms of the feasibility of apps, with little research being conducted on the attractiveness of apps for target users. The largest gap identified in the literature was evidence-based testing into the efficacy of apps, as the rapidity of technological advancement presents challenges for researchers in terms of testing newly developed apps [23].

### Aims of This Research

The aims of this research are to bridge research gaps by developing an mHealth self-management support intervention to help students engage with learning and ensuring the intervention is evidence-based. Therefore, the primary aim of the proposed research is to examine the effectiveness of an mHealth intervention, ReZone, in reducing emotional and behavioral difficulties in young people to support engagement with school. The secondary aims are to examine the effectiveness of ReZone in improving self-management, well-being, and health-related quality of life in young people.

## Methods

### Participants and Procedures

The sample size calculation was performed using the clustersampsi command [24] in Stata 12 (StataCorp LLC). The primary outcome measure is behavioral difficulties [25].

A minimum of 12 clusters (classes) of 10 students will be recruited. This is based on a mean effect size for universal classroom management programs of *d*=0.80 and an intraclass correlation coefficient (ICC) of 0.05 [26], a mean (standard deviation) in the intervention arm of 2.42 (2.05) indicating that behavioral difficulties are in the nonclinical range and a mean in the control arm of 5.13 (2.74) indicating that behavioral difficulties are in the clinical range [25], an average cluster size of 10 students and a coefficient of variation of cluster sizes of 0.42 [27], and a correlation between before-and-after measurements of 0.5. As the sample is being stratified by alternative provision versus mainstream school, 3 classes of each type per arm are being recruited across South East England.

All students aged 10 to 15 years in the schools taking part in the project will be eligible to participate. To ensure matched ages across the conditions, 5th grade students (aged 10 to 11 years) will be recruited from mainstream schools; the alternative provision schools have classes with mixed ages, and, therefore, students aged 10 to 15 years will be recruited from these schools. Students will be informed about the study by schools, which will disseminate information sheets and consent forms. Consent will be recorded in writing from parents with assent recorded from young people. Participants will be assigned a unique number and their data will only be identified by this number; consent forms and contact details will be stored securely and separately from anonymized study data. Participant lists of all classes and students will be obtained from schools.

An independent trials unit will randomize classes to management as usual or ReZone stratified by school type (alternative provision vs mainstream) using random sequence generation after classes have been recruited and baseline measures assessed. To reduce the predictability of randomization, classes will be randomized in blocks of 2, and allocation will be implemented via a secure online portal.

Management as usual was deemed an appropriate comparator as there is no routinely used mHealth intervention in classes when students feel overwhelmed. The trial will be open label.

Research assistants will attend classes, and students will complete baseline paper questionnaires (this took place from February to April 2017). Classes will then begin management as usual or download and start using ReZone. Research assistants will attend classes to collect follow-up paper questionnaires 3 months later (this took place from April to June 2017).

A favorable opinion has been received from University College London (UCL) Research Ethics (number 7969/001), and the study is adhering to relevant ethical guidelines from the British Psychology Society [28]. The study is sponsored by UCL, and trial materials will be made available to the sponsor for auditing upon request. The trial is registered with ISRCTN [ISRCTN13425994], and the protocol is reported in line with Standard Protocol Items: Recommendations for Interventional Trials guidelines [29]. ReZone is reported according to guidelines for reporting mHealth interventions [30].

### Intervention

#### Infrastructure (Population Level)

There are 3.6 million school students aged 10 to 15 years old in the United Kingdom [31]. The rapid increase of teledensity—56% of children aged 8 to 12 years [32] and 88% of adolescents aged 13 to 17 years own or have access to a mobile phone [33]—combined with the increase in access to a tablet or e-reader—58% of adolescents have access to a tablet computer and 35% of teachers have access to a tablet computer or e-reader in the classroom (compared to only 20% of teachers in 2012) [34]—allows ReZone to reach a large population.

#### Technology Platform

ReZone was made for Apple iOS, Google Android, and Google ChromeBook. It was developed using HTML, Cascading Style Sheets, and JavaScript and using Phonegap to convert into iOS and Android apps. The server querying was performed using hypertext preprocessor (PHP) and a MySQL database. The server is a Dell PowerEdge R210 with Quad Core 2.40 Ghz Linux-based dedicated server with 1 TB bandwidth. The ChromeBook app is kept as a packaged HTML app.

#### Interoperability and Health Information System Context

As ReZone is being used in a classroom setting, it is not necessary to connect or interact with national or regional health information systems at this stage.

#### Intervention Delivery

Students are able to download the ReZone app from the iTunes, Google Play, and Windows app stores. Students can then use the app as much as they like, using the tools on the platform as well as being able to download and email the flat graphics of tools without personal data. Students can use ReZone on their own accord or be directed by teachers to use it during classes at times the student is becoming overwhelmed.

#### Intervention Content

The app aims to help students manage their emotional well-being in the classroom by encouraging them to refocus if they are feeling angry, stressed, or anxious. ReZone contains a series of tools designed to improve concentration, help relieve stress, and help students to reflect and think through problems logically. Consultations with staff from alternative provision schools were conducted where researchers discussed the therapeutic tools already being used within the school and which elements of the tools they felt were important. ReZone was then reviewed as part of the App Approval Application panel at the Anna Freud National Centre for Children and Families. This feedback, along with mentalization-based therapy, cognitive behavioral therapy (CBT), mindfulness breathing, and evidence-based training such as attention bias modification training (ABMT) aided the design of ReZone.

#### Functions

**Figure 1:**
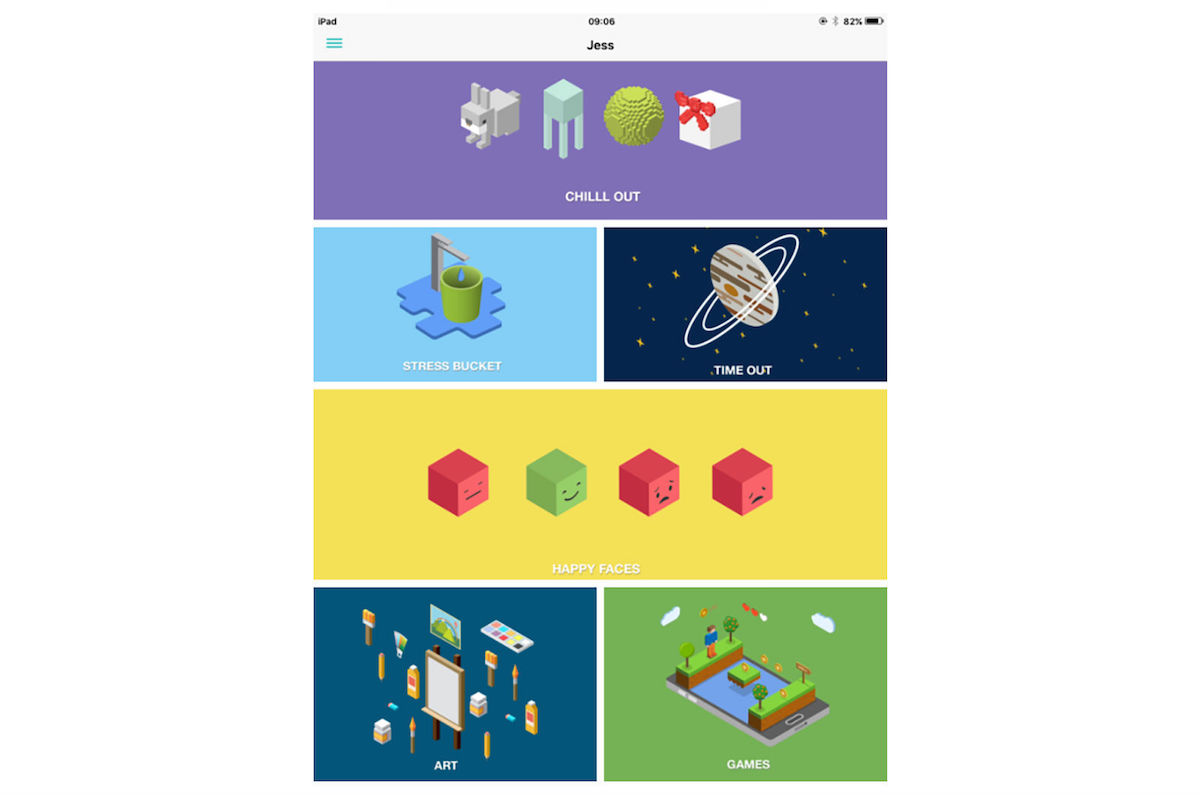
ReZone home page.

There are 6 functions of Rezone (stress bucket, chill out, art therapy, timeout, happy faces, and games) (Figure 1) which are based in CBT, mindfulness, and ABMT.

**Figure 2:**
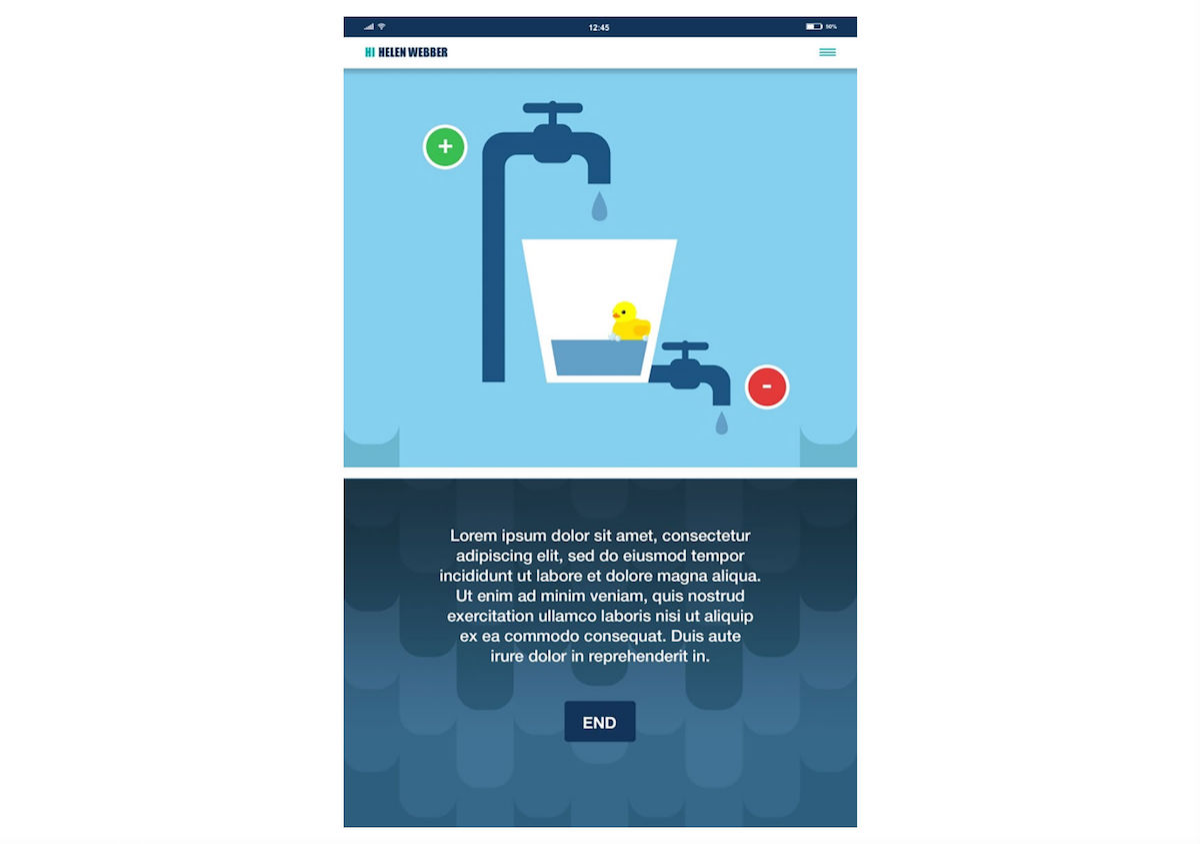
ReZone stress bucket (before).

**Figure 3:**
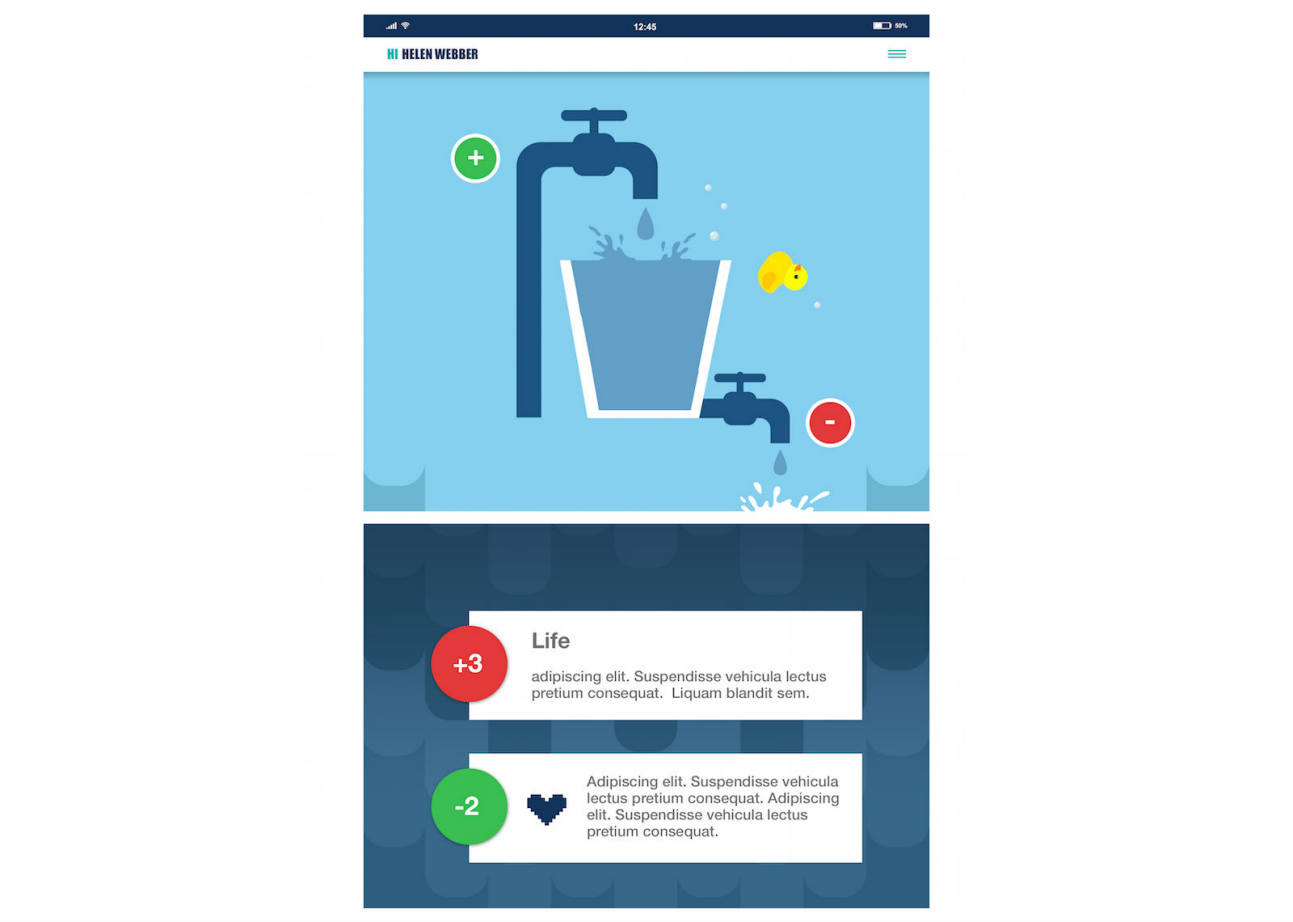
ReZone stress bucket (after).

**Figure 4:**
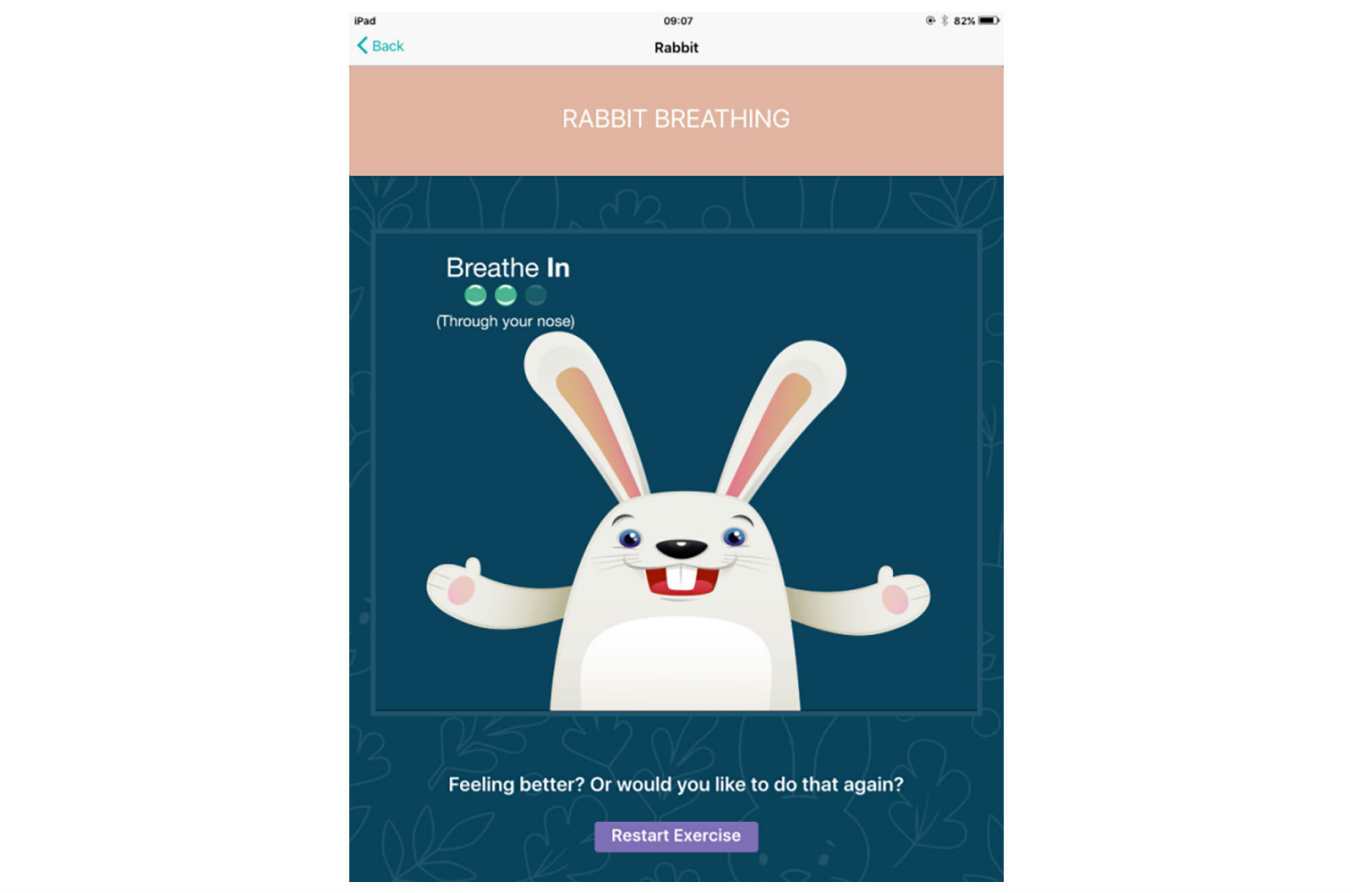
ReZone chill out function; breathing exercise.

- Stress bucket (Figures 2 and 3): The stress bucket lets the user add any stressors that they are experiencing to a bucket. They are then able to introduce activities that help them cope with each stressor. They can see the water in the bucket rise and fall as they add and relieve stressors. If the bucket reaches 50 stress points, it will overflow.
- Timeout: Timeout asks the user to think through a time when they have felt stressed, angry, or upset. The user then works through on the app all the events that led up to feeling this way and what happened afterwards. The user can also think through what they could have done differently to help the situation as a behavioral plan. The visualization is a rocket, and each thought process creates a cloud.
- Chill out (Figure 4): Chill out uses breathing to help the user calm down and relax. Each chill out activity is based around an object or animal (rabbit, jellyfish, ball, or square) using breathing in different ways.
- Art therapy: The user can choose between a castle, dinosaur, fish, goat, heart, helicopter, unicorn, rocket, footballer, sea, or turtle to color in. There is a range of colors and utensils to complete the drawing.
- Happy faces: The user is given 30 seconds to find as many happy faces as they can among other faces depicting negative emotions.
- Game: There is game of Balloon Blast on the app. The user taps the screen to move the balloon up, trying to avoid all the obstacles, as hitting one will mean the game is over. This is a game created to provide a break or reward for the user in between the other features.

### Theory

#### Cognitive Behavioral Therapy

The stress bucket and timeout are based on the mechanism of CBT, a widely used technique looking at how a person thinks about a situation and how this affects the way they act [35]. It has been shown to successfully treat many conditions including depression and anxiety in young people in a variety of settings and formats when delivered through a digital platform [36].

#### Mindfulness

Chill out and art therapy are based on the concept of mindfulness [37,38]. This can be defined as the self-regulation of attention and orientation so that it is maintained on immediate experiences, allowing for increased recognition of mental events in the present moment, curiosity, openness, and acceptance. Mindfulness has been shown to have many benefits for children and young people [38]. For example, evidence suggests that mindfulness can improve the mental, emotional, social, and physical health of young people. It can also increase the ability to manage behavior and emotions while reducing stress and anxiety [39,40].

#### Attention Bias Modification Training

Happy faces uses techniques from ABMT, which aims to alter cognitive biases that selectively focus on negative emotional cues in the environment [41]. It has been shown that if children search for happy faces among angry faces, greater posttraining attention bias toward happy faces is evident. It also has significantly reduced clinician-rated anxiety [42].

### Usability and Content Testing

Designing the app began with formative research with alternative provider schools to elicit feedback about the current use of tools when feeling angry, stressed, or anxious and current mobile phone and tablet use in and outside of the classroom. Students were also recruited to test prototypes and provide feedback on the usability, content, and design of the app. We will also be conducting focus groups and interviews with teachers and students at the end of the trial to gather further feedback on usability and content.

### User Feedback

In the initial feedback on the designs, students reported that the tools were useful and easy to understand, and they appreciated the ability to have a range of tools in one place. Students offered their opinions on the design and wording of the app for their age range and on how best to share their data with their teachers. Given the existing user feedback, it is not anticipated that ReZone will cause distress. However, if students were to experience distress from using ReZone, it would be recorded as a serious adverse event, the ethics committee and sponsor would be notified, and the trial would be stopped if necessary.

### Access of Individual Participants

ReZone is designed to be used in school settings with 10- to 15-year-old students, who commonly have access to tablets within school. Access could be affected by socioeconomic status due to the cost of a mobile phone or tablet to families or schools.

### Cost Assessment

ReZone is free to download from the relevant app stores and, therefore, the cost of the intervention to the school is for Internet access in order to download and run the app and the cost of the tablets and/or phones. The financial strategy of ReZone and other digital products in our center is to cover ongoing technical maintenance of the app and systems through costed training for schools and mental health services in using specific products such as ReZone, using digital products in these settings in general, and developing and evaluating in-house digital products.

### Adoption Inputs and Program Entry

ReZone has information points and examples throughout the app and the 6 individual functions so that the intervention use is clear to both students and staff. Students were recruited to give feedback on the information points and usability of the app. Additional training will not be provided as ReZone has been developed to be used as a standalone intervention.

### Limitation for Delivery at Scale

Access to mobile phones and tablets at school is the main limitation for delivery at scale, but this is more applicable to mainstream schools than alternative provider schools where mobile phones and tablets are routinely used during learning.

### Contextual Adaptability

The functionality of ReZone applies to a range of school settings. The intervention is free and easily downloaded from various app stores; it is not limited to specific users or geographical localities. It will be important to examine how best to adapt ReZone for other cultures, groups, and settings as part of other projects.

### Replicability

Screenshots of content are included in Figures 1 to 4 to provide information and further context for replicating the study.

### Data Security

Students are asked to enter their name, school, and date of birth each time they start using the app. The app combines the 3 sets of data encryption before submitting to the server using an Internet connection. These data are encrypted again at server level using PHP and saved in encrypted format inside a MySQL database with a unique identifier. Name, school, and date of birth are not stored as plain text in the database. If the data were to be intercepted, the data would be meaningless because they are encrypted before leaving the app. With regard to the ChromeBook app, a SHA256 Secure Sockets Layer certificate will be added to encrypt the data further. The server itself has physical and software-based security such as cameras around the hardware, daily backups, and a firewall.

### Compliance With National Guidelines or Regulatory Statutes

The tools used on ReZone are based on the relevant supporting evidence base for mentalization-based therapy, CBT techniques, and ABMT. They are evidence-informed tools that have been previously created and align with relevant recommendations, such as CBT for anxiety as recommended by National Institute for Health and Care Excellence guidelines [43].

### Fidelity of the Intervention

Activity data are stored on the app and will be reviewed to determine how long students spend on ReZone, which tools they use the most, and for how long they use each tool. Metrics of participant engagement with the intervention will also be measured by teachers on self-reported forms. Teachers will record fidelity (eg, the number of times ReZone is used per class, the number of students using ReZone), class disruption, other SEAL interventions being used, and length of time to reinstate student to the class. Participants can also print off activities completed on the app to show teachers or family members.

### Measures

#### Demographic Characteristics

Age, gender, and ethnicity will be self-reported by young people at baseline. Ethnicity will be captured using the categories from the 2001 Census. Special educational needs will be obtained from school records.

#### Emotional and Behavioral Difficulties

To measure emotional and behavioral difficulties, the 16-item Me and My School (M&MS) [25] will be used. The M&MS measure comprises 2 subscales assessing emotional difficulties (10 items; eg, “I feel lonely,” “I worry a lot”) and behavioral difficulties (6 items; eg, “I lose my temper,” “I break things on purpose”). Young people respond to all items on a 3-point scale from 0=never to 2=always. The M&MS has been used in previous studies and demonstrated reliability and validity [25]. Moreover, clinical cut-off scores have been established of 10 or more for emotional difficulties and 6 or more for behavioral difficulties.

#### Mental Well-Being

To measure mental well-being, the 7-item Short Warwick-Edinburgh Mental Well-being Scale (SWEMWBS) [44] will be used. The SWEMWBS measures positive mental well-being (eg, “I’ve been feeling useful,” “I’ve been feeling relaxed”). Young people respond to all items on a 5-point scale from 1=none of the time to 5=all of the time. The SWEMWBS has been used in previous studies and demonstrated reliability and validity [44].

#### Self-Management

To measure self-management, the 6-item self subscale of the Youth Empowerment Scale–Mental Health (YES-MH) [45] will be used. The self subscale captures empowerment to self-manage mental health difficulties (eg, “I know how to take care of my mental or emotional health,” “I feel my life is under control”). Young people respond to all items on a 5-point scale from 1=never or almost never to 5=always or almost always. The YES-MH has been used in a previous study and demonstrated reliability and validity [45].

#### Health-Related Quality of Life

To measure health-related quality of life, the 6-item EuroQol 5-Dimension–Youth (EQ-5D-Y) [46] will be used. The EQ-5D-Y captures current health states regarding 5 specific health domains (eg, mobility, self-care) on a 3-point scale from 1=no problems to 3=a lot of problems and global health on a visual analog scale. The EQ-5D-Y has been used in previous studies and demonstrated reliability and validity [46].

### Analytic Strategy

Data will be entered into an Excel (Microsoft Corp) spreadsheet by research assistants with a random 20% double-entered for cross-checking. Data will only be stored on the organization’s secured servers, accessible only by members of the research team. Data will be analyzed using Stata 12 (StataCorp LLC) in order to examine the effectiveness of ReZone in reducing emotional and behavioral difficulties and improving self-management, well-being, and health-related quality of life in young people in need of targeted support to engage with learning. Three models will be tested for each of the outcome variables with time nested within students within classrooms. In model 0, the null model without predictors will be computed to examine change in outcome (eg, behavioral difficulties) over time, and the ICC will be calculated. In model 1, the association patient-level grand mean centered age, ethnicity, and special educational needs will be entered as a level-1 predictor. In model 2, the condition (ReZone vs management as usual) will be entered. School-type (ie, alternative provision vs mainstream) will be entered as a fixed effect. The likelihood ratio test will be used to compare the fit of subsequent models. An intention-to-treat analysis will be performed with last-item carried forward imputation.

## Results

Funding for the trial has been secured from University College London (UCL). Baseline data collection started in February 2017 and ended in April 2017. Follow-up data collection started in April 2017 and ended in June 2017. Data analysis and write-up will be completed by December 2017.

Any updates to this protocol will be published, and the findings of the proposed research will be submitted for publication in a peer-reviewed journal by the current authors in line with International Committee of Medical Journal Editors’ guidelines. The research team will also disseminate findings to the participating schools, the study sponsor, and relevant conferences. A public summary of the findings will be available on our organization’s website (www.ucl.ac.uk/ebpu).

## Discussion

The trial and its findings will develop an evidence-based mHealth self-management support intervention to help students engage with learning and manage their emotions. The findings will contribute to the growing use of technology to support children and young people with their mental health. Anticipated limitations of the proposed research include the fidelity of the intervention, assessed through teacher self-report and activity data; the content of management as usual, as different classes in this arm may use a variety of different social and emotional learning interventions despite not using ReZone, assessed through teacher self-report; and attrition, addressed by designing ReZone with young people to ensure it is usable and through using existing questionnaires that balance length with capturing the necessary range of relevant outcomes. Notwithstanding the above limitations, the proposed research will provide evidence as to whether ReZone is effective in helping young people to self-manage when feeling overwhelmed.

## Abbreviations

ABMT: attention bias modification training
CBT: cognitive behavioral therapy
EQ-5D-Y: EuroQol 5-Dimension–Youth
ICC: intraclass correlation coefficient
M&MS: Me and My School
PHP: hypertext preprocessor
SEAL: social and emotional aspects of learning
SWEMWBS: Short Warwick-Edinburgh Mental Well-being Scale
UCL: University College London
YES-MH: Youth Empowerment Scale–Mental Health
