# Correction: An App to Help Young People Self-Manage When Feeling Overwhelmed (ReZone): Protocol of a Cluster Randomized Controlled Trial

**DOI:** 10.2196/10018

**Published:** 2018-03-16

**Authors:** Julian Edbrooke-Childs, Jaime Smith, Jessica Rees, Chloe Edridge, Ana Calderon, Felicity Saunders, Miranda Wolpert, Jessica Deighton

**Affiliations:** ^1^ Evidence Based Practice Unit University College London and Anna Freud National Centre for Children and Families London United Kingdom; ^2^ Anna Freud National Centre for Children and Families London United Kingdom

The authors of “An App to Help Young People Self-Manage When Feeling Overwhelmed (ReZone): Protocol of a Cluster Randomized Controlled Trial” (JMIR Res Protoc 2017;6(11):e213) would like to add the following Acknowledgments section as it is important funding information:

The research was supported by the National Institute for Health Research (NIHR) Collaboration for Leadership in Applied Health Research and Care (CLAHRC) North Thames at Bart’s Health NHS Trust. The views expressed are those of the authors and not necessarily those of the NHS, the NIHR or the Department of Health.

The corrected article will appear in the online version of the paper on the JMIR website on March 16, 2018, together with the publication of this correction notice. Because this was made after submission to PubMed or PubMed Central and other full-text repositories, the corrected article also has been re-submitted to those repositories.

